# Mesenteric Lymphadenitis Due to COVID-19 in an Adult

**DOI:** 10.7759/cureus.15897

**Published:** 2021-06-24

**Authors:** Haris Iftikhar, Mavia Najam, Mujeeb U Rehman

**Affiliations:** 1 Emergency Medicine, Hamad Medical Corporation, Doha, QAT; 2 Medicine, Nishtar Medical University, Multan, PAK

**Keywords:** acute appendicitis, acute abdomen, mesenteric lymphadenitis, covid- 19, sars-cov-2

## Abstract

Coronavirus disease 2019 (COVID-19) is an infectious disease that can present with a wide range of symptoms. Abdominal pain is less common than other symptoms but is more frequent among patients with severe disease. Various abdominal imaging findings are described in the literature for children and adults with COVID-19 infection. Mesenteric lymphadenopathy is reported in pediatric patients with COVID-19 gastrointestinal infection. It is very rarely reported in the adult population.

We report a case of an adult male with multiple risk factors, who presented with severe abdominal pain and tenderness in the right inguinal fossa. He was evaluated for differential diagnosis of acute appendicitis, renal colic, diabetic ketoacidosis (DKA), and COVID-19. His investigations showed normal laboratory tests and a normal chest radiograph. His CT abdomen showed a normal appendix and multiple prominent mesenteric lymph nodes. His COVID-19 PCR was positive. He was discharged after pain relief with home isolation instructions and symptomatic management.

Our case represents an atypical clinical presentation of COVID-19 infection in many ways. His laboratory investigations were not suggestive of COVID-19. Our patient’s abdominal imaging findings represent a rare association of COVID-19 with mesenteric lymphadenitis in adults. The clinical course of our patient was smooth after discharge and he did not develop any complications of COVID-19 despite multiple risk factors. Our case reminds the significance of keeping broad diagnostic differentials in the emergency department. Although mesenteric lymphadenitis is often a self-limiting condition affecting children and young adults, it is the most frequent alternative diagnosis of acute appendicitis and intussusception. Mesenteric lymphadenitis can be the sole atypical presentation of COVID-19 in adults. Atypical presentations are not uncommon due to the scarcity of data on this evolving disease.

## Introduction

Coronavirus disease 2019 (COVID-19) is an infectious disease caused by a positive-sense single-stranded genomic RNA virus. COVID-19 may be asymptomatic or it can cause a wide range of symptoms and life-threatening sepsis [1]. Common symptoms include fever, dry cough, shortness of breath, fatigue, myalgias, nausea/vomiting or diarrhea, headache, weakness, rhinorrhea, Anosmia, and ageusia [2-3]. Abdominal pain is found at a lower rate than other symptoms but is more frequent among patients with severe disease [2]. Mesenteric lymphadenopathy on abdominal imaging is often an incidental finding and can be a benign condition due to infectious etiology. It is reported in pediatric patients with COVID-19 infection. It is very rarely reported in the adult population. It is named as Jammu and Kashmir sign (JK sign) in one case report [4]. We report a case of an adult male with multiple comorbidities, who presented with mesenteric lymphadenitis as an atypical presentation of COVID-19.

## Case presentation

A 47-year-old male with a past medical history of poorly controlled diabetes, hypertension, and hyperlipidemia presented with severe colicky right lower abdominal pain and subjective fever for two days. He denies radiation of pain, association with nausea or vomiting, change in urinary or bowel movements. He denies sore throat, runny nose, change in smell or taste, fatigue, myalgias, and shortness of breath. He works as a gardener and denies any sick contacts. His home medications include enalapril, atorvastatin, metformin, insulin aspart, and glargine.

Physical exam showed a temperature of 36.8°C, respiratory rate of 17 breaths/minute, BP of 114/77 mmHg, and saturation of 98% on room air. His abdominal examination shows a soft abdomen with tenderness in the right inguinal fossa but no rebound tenderness or signs of peritonitis. His testicular exam was normal. The rest of his systemic examination was also normal. His bedside random glucose was 23.5 mmol/l. He was evaluated for differential diagnosis of acute appendicitis, renal colic, diabetic ketoacidosis (DKA), and COVID-19. He received one liter of normal saline, six units of subcutaneous insulin regular, and one gram of paracetamol for initial pain relief.

**Figure 1:**
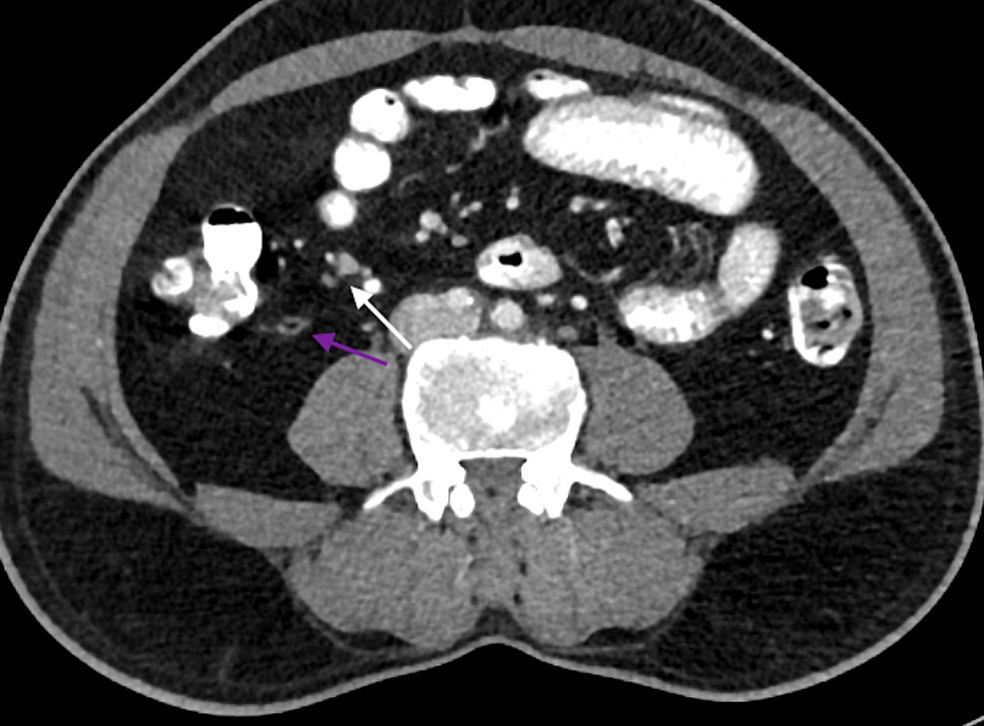
Contrast-enhanced CT abdomen axial view showing normal appendix with intraluminal air (purple arrow). The white arrow shows enlarged mesenteric lymph nodes.

**Figure 2:**
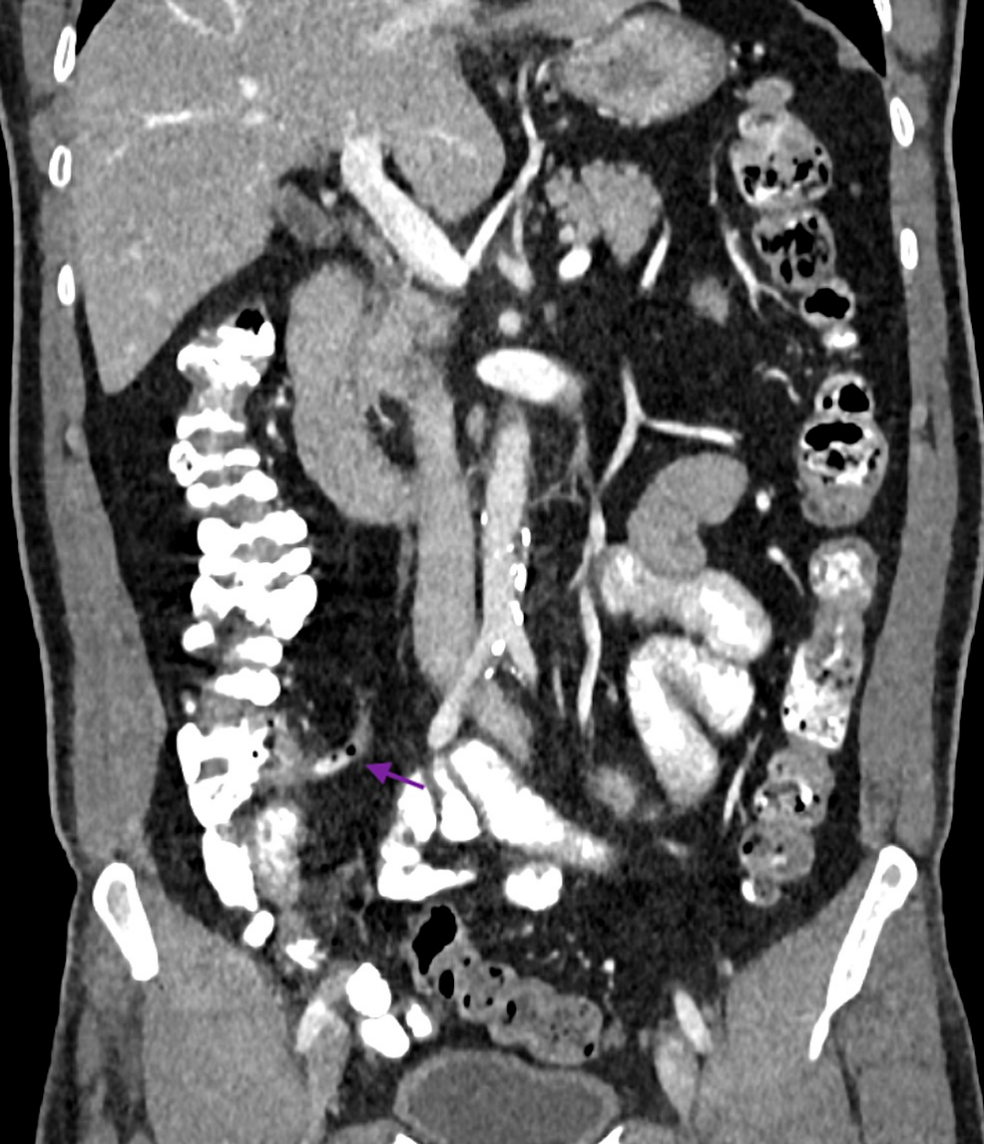
Coronal section of contrast-enhanced CT abdomen showing normal appendix (purple arrow) with intraluminal air and contrast.

His investigations showed normal complete blood count, comprehensive metabolic panel, C-reactive protein, beta-hydroxybutyrate, D-dimer, ferritin, lactate dehydrogenase, lactic acid, lipase, procalcitonin, urine dipstick, and a normal chest radiograph. His CT abdomen showed a normal appendix with no peri-appendiceal fat standing. There were multiple prominent mesenteric lymph nodes in the ileocolic region with subtle fat stranding, likely to represent mesenteric lymphadenitis (Figures 1, 2). His COVID-19 PCR (nasal and pharyngeal swab) came out to be positive. According to institutional guidelines, he was discharged after pain relief with home isolation instructions and symptomatic management. His follow-up showed he recovered completely without any complications.

## Discussion

Atypical presentations are possible in COVID-19. Common atypical presentations include asymptomatic patients, CT imaging-negative, and re-detectable positive patients [5]. In one retrospective study in elderly patients with COVID-19, the most common atypical presentations were falls, decreased mobility or generalized weakness, and delirium [6]. Our case represents an atypical and unique clinical presentation of COVID-19 infection in many ways. His laboratory investigations were not suggestive of COVID-19. Common laboratory abnormalities in COVID-19 include lymphopenia, elevated inflammatory markers (e.g., erythrocyte sedimentation rate, C-reactive protein, ferritin, tumor necrosis factor-α, IL-1, IL-6), and abnormal coagulation parameters (e.g., prolonged prothrombin time, thrombocytopenia, elevated D-dimer, low fibrinogen) [7]. Our patient’s abdominal imaging findings represent a rare association of COVID-19 with mesenteric lymphadenitis in adults. In literature, abdominal ultrasonography in critically ill children with COVID-19 can show mesenteric lymphadenopathy, hepatomegaly, nephromegaly, gallbladder wall edema, ascites, and intestinal inflammation. CT showed fluid-filled small bowel loops, mural thickening of the terminal ileum, diffuse lymphadenopathy, and moderate ascites [8]. The abdominal imaging in adults with COVID-19 showed enteritis or mesenteric ischemia. Patients with mild infectious colitis/enteritis may have bowel wall thickening, mesenteric fat stranding, or a small amount of ascites. Some patients present with small bowel obstruction and pancreatitis [9]. The clinical course of our patient was smooth after discharge and he did not develop any complications of COVID-19 despite multiple risk factors. Common complications reported in the literature include pneumonia, acute respiratory distress syndrome, acute liver injury, cardiac injury, including troponin elevation, acute heart failure, dysrhythmias, and myocarditis; venous and arterial thromboembolic events, acute kidney injury, neurologic manifestations, and shock [1].

Our case reminds the significance of keeping broad diagnostic differentials in the emergency department. In this COVID era, emergency physicians should keep themselves up-to-date with the possible atypical presentations of this evolving disease. Although mesenteric lymphadenitis is often a self-limiting condition affecting children and young adults, it is the most frequent alternative diagnosis of acute appendicitis and intussusception. Abdominal pain in this condition usually disappears in 2-3 weeks. Supportive care with hydration and pain relief is the mainstay of treatment. Patients can be discharged home after explaining the diagnosis and reassurance [10].

## Conclusions

Mesenteric lymphadenitis, a benign self-limiting condition in children, can be the sole atypical presentation of COVID-19 in adults. Atypical presentations are not uncommon due to the scarcity of data on this evolving disease. Emergency physicians should keep broad differential diagnoses so that appropriate investigations can be done.
